# Lymphomatous infiltration of the kidney in a patient with Waldenstrom’s macroglobulinemia 

**DOI:** 10.5414/CNCS110756

**Published:** 2022-12-15

**Authors:** Prasanth Ravipati, Lihong Bu, Zohar Sachs, Patrick H. Nachman

**Affiliations:** 1 Department of Internal Medicine, Division of Nephrology, University of Nebraska Medical Center, Omaha, NE,; 2 Department of Pathology and Laboratory Medicine,; 3 Department of Internal Medicine, Division of Hematology, Oncology, and Transplantation, and; 4 Department of Internal Medicine, Division of Nephrology and Hypertension, University of Minnesota Medical Center, Minneapolis, MN, USA

**Keywords:** lymphoma, paraproteinemia, tubulointerstitial disease, acute kidney injury, SARS-CoV-2

## Abstract

Kidney disease can be an initial presentation or a chronic manifestation of plasma cell dyscrasias. Here, we describe a rare presentation of kidney disease driven by lymphomatous infiltration of the kidney in a patient with Waldenstrom’s macroglobulinemia (WM). A 70-year-old female with an 8-year history of WM (IgM, κ) was referred for declining renal function. Prior to presentation, she had stable WM disease without evidence of worsening disease burden. She had been previously hospitalized with SARS-CoV-2 infection and acute kidney injury (AKI). Her serum creatinine (sCr) peaked at 3.7 mg/dL (baseline 0.9 mg/dL) but recovered to 1.1 mg/dL by the time of discharge. Two months after discharge, her sCr increased to 1.9 mg/dL, and she had new proteinuria of 1.5 g/day. Kidney biopsy showed lymphomatous infiltration of the interstitium without glomerular involvement. Treatment with rituximab and bendamustine resulted in an improvement in renal function (sCr 1.4 mg/dL). WM is an uncommon hematologic malignancy, and extramedullary involvement, including renal involvement, is rare. This case emphasizes the importance of surveillance for kidney dysfunction in patients with plasma cell dyscrasias, even if patients appear to have stable lymphoproliferative disease.

## Introduction 

Plasma cell dyscrasias include a wide spectrum of diseases such as multiple myeloma, amyloidosis, and monoclonal gammopathy of undetermined significance (MGUS), or of renal significance (MGRS). Kidney disease can be an initial presentation or a chronic manifestation of plasma cell dyscrasias, and pathologic changes can occur in the glomerulus as well as in the tubulointerstitium [1]. However, kidney infiltration by malignant lymphocytes is uncommon. The aim of this case report is to illuminate a rare presentation of kidney disease driven by lymphomatous infiltration of the kidney in a patient with Waldenstrom’s macroglobulinemia (WM). WM, a subtype of lymphoplasmacytic lymphoma (LPL), is a plasma cell dyscrasia characterized by elevated levels of circulating monoclonal immunoglobulin M (IgM) paraprotein [2]. 

## Case report 

A 70-year-old female with a history of WM was referred to nephrology for declining renal function. She had been diagnosed with WM (immunoglobulin M of κ light chain type) 8 years prior to presentation, when she presented with fatigue and anemia with a hemoglobin of 9.7 g/dL. A serum protein electrophoresis showed an M spike of 1.5 g/dL. After bone marrow biopsy confirmed the diagnosis of WM, she underwent treatment with single-agent rituximab (per patient preference), resulting in normalization of her hemoglobin and fatigue. She had a relapse of WM 3 years later, which was heralded by anemia and fatigue. Subsequently, she was started on maintenance rituximab (again per patient preference to avoid more aggressive therapy), which was continued for 2 years. After discontinuation of rituximab, she had stable disease with Hgb in the 11.5 – 13.0 g/dL range, and the M spike ranged between 1.0 and 1.6 g/dL. 

She contracted SARS-CoV-2 in 2020, complicated by mental status changes, acute kidney injury, hospitalization, and she required non-invasive ventilation and vasopressor support for shock. Her baseline serum creatinine (sCr) prior to admission was 0.8 – 0.9 mg/dL, and she had no prior history of kidney disease. During her 25-day hospitalization, her sCr peaked at 3.7 mg/dL but later improved to 1.1 mg/dL over one month following discharge. However, at a post-hospitalization follow-up a month later, her sCr had increased to 1.4 mg/dL. A repeat sCr 3 weeks later showed continued rise to 1.9 mg/dL. Urinalysis was positive for albuminuria but was without hematuria or pyuria. A 24-hour urine protein collection confirmed 1.5 g/day of proteinuria. 

**Figure 1:**
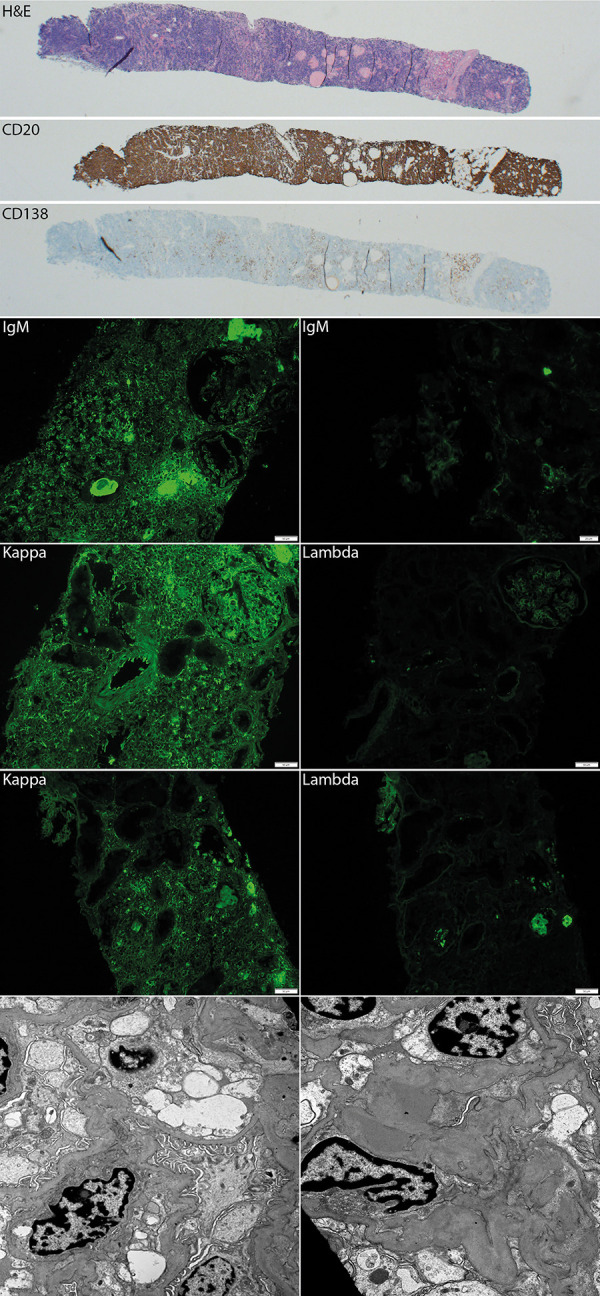
Representative images of renal biopsy findings. Light microscopy shows diffuse dense interstitial lymphoplasmacytic infiltrate (H & E stain, × 200) without evident glomerular lesions or tubulitis. The interstitial infiltrate is composed mainly of CD20-positive B lymphocytes (× 200) with admixed CD3-positive T lymphocytes (not shown) and CD138-positive plasma cells (× 200). By immunofluorescence, the interstitial inflammatory cells are predominantly positive for IgM (left × 200) and κ light chain (× 200), and negative for λ light chain (× 200). There is no significant glomerular staining (IgM, right, × 400). Tubular casts are positive for both κ and λ light chains. Electron micrographs reveal glomerular basement membrane with a trilaminar structure and a mean of thickness within normal range (left 9280X). No immune complex or paraprotein-related deposits were seen. Rare hyaline type mesangial deposits are noted (right × 13,900). There is less than 50% podocyte foot process effacement. Endothelial cells show preserved fenestration.

Kidney biopsy light microscopy revealed diffuse, dense interstitial lymphoplasmacytic infiltrate without significant tubulitis. The inflammatory cells stained predominantly positive for CD20, with κ predominance, and negative for CD3, CD5, and CD10. Stains for CD138 showed occasional loose aggregates of κ-predominant plasmacytoid cells (Figure 1). 13 glomeruli were identified, 9 of which were globally sclerotic. The non-sclerotic glomeruli were normocellular without mesangial matrix expansion. Glomerular basement membranes were smooth and single contoured, and there were no crescents, thrombi, or segmental lesions. Three glomeruli were present for immunofluorescence. The interstitial infiltrate was strongly positive for IgM and κ light chain, but negative for λ light chain. There was sparse granular mesangial staining for IgM, C3, κ, and λ. No tubular basement membrane staining was present. By electron microscopy, 3 glomeruli were present, 2 of which were globally sclerotic. Normal cellularity and mesangial regions were confirmed, and no immune complex or paraprotein-related deposits were visualized. The glomerular basement membranes showed no ultrastructural abnormalities. There was minimal foot process effacement present. Examination of the tubulointerstitial compartment confirmed interstitial inflammation, and no tubular basement membrane deposits were seen. These findings were consistent with extramedullary extension of WM/LPL. 

Bone marrow biopsy confirmed persistence of WM/LPL involving 25% of her bone marrow. A positron emission tomography scan showed progression of previously stable lymphadenopathy. Treatment with bendamustine and rituximab was initiated, resulting in an improvement in creatinine from 1.9 to 1.4 mg/dL after 3 of 6 planned cycles of treatment. 

## Discussion 

WM is an uncommon hematologic malignancy with an incidence of 1,500 new cases per year in the United States. Generally, WM has an indolent course, but there is phenotypic variation ranging from asymptomatic to patients with severe disease [3]. Treatment can range from observation to initiation of chemotherapeutics, such as bendamustine or cyclophosphamide, in combination with anti-CD20 therapy. Extramedullary and extranodal involvement, particularly infiltration of the kidney, is rare. In a retrospective analysis from the Mayo Clinic of a cohort of 1,363 patients with WM and other IgM secreting B-cell lymphoproliferative disorders, 57 patients underwent kidney biopsy, of whom 47 had monoclonal gammopathy-related kidney lesions. Only 4 patients were found to have lymphomatous infiltration of the kidney [4]. In the case presented here, the differential diagnosis for renal insufficiency included acute tubular injury, paraprotein-related disease in the setting of WM, possibly interstitial nephritis related to proton pump inhibitor that was initiated during hospitalization, or an undefined sequela of SARS-CoV-2 infection. 

The chronologic association between the progression/reactivation of WM after the SARS-CoV-2 infection is intriguing. Prior to the infection, she had stable disease by imaging and laboratory evaluation for 2 years, and no sign of disease progression or transformation at the most recent hematology evaluation prior to her illness with SARS-CoV-2. She had not been experiencing fevers, weight loss, or night sweats. Her laboratory parameters showed stable Hgb, stable M Spike at 1.0 g/dL, and normal lactate dehydrogenase. Additionally, her serum creatinine was at a baseline of 0.9 mg/dL just prior to her hospitalization for SARS-CoV-2. Viral infections have been described to be involved in the pathogenesis of lymphomas [5], in particular human herpes virus 4 (HHV-4) and human herpes virus 8 (HHV-8) can cause B-cell lymphomas. The mechanisms are not completely clear but are related to generation of proteins that inhibit lymphocyte apoptosis. It has been postulated that HHV-4 could be involved in the transformation (Richter) of chronic lymphocytic leukemia to diffuse large B-cell lymphoma or Hodgkin’s disease [5, 6]. To date, there is no evidence of SARS-CoV-2 resulting in progressive disease in indolent lymphomas, however, there has been a reported case of remission of lymphoma after SARS-CoV-2 infection [7]. 

## Conclusion 

Although lymphomatous infiltration of the kidney in WM is a rare event, it should be considered in patients presenting with abnormal renal function. The long-term effects of SARS-CoV-2 infection in patients with indolent lymphomas are not yet known, but it is noteworthy that our patient developed lymphomatous kidney involvement after initial recovery of acute kidney injury in the setting of severe SARS-COV-2 infection. Whether SARS-CoV-2 infection coupled with a host’s immune response can alter disease course of lymphoma has not yet been elucidated, but as more data is gathered over time, this hypothesis may be studied. Overall, this case emphasizes the importance of surveillance for kidney dysfunction in patients with plasma cell dyscrasias, even if patients appear to have stable lymphoproliferative disease. 

## Funding 

The authors received no funding for this work. 

## Conflict of interest 

The authors declare no conflict of interest.
